# Physical Functional Impairment in Breast Cancer Patients: A Cross-Sectional Expert Survey

**DOI:** 10.7759/cureus.57364

**Published:** 2024-03-31

**Authors:** Suman Mehra, Pragya Kumar, Abhishek Soni

**Affiliations:** 1 Department of Physiotherapy, Amity Institute of Health Allied Sciences, Amity University, Noida, Noida, IND; 2 College of Physiotherapy, Pandit Bhagwat Dayal Sharma Post Graduate Institute of Medical Sciences (PGIMS), Rohtak, IND; 3 Department of Radiation Oncology, Pandit Bhagwat Dayal Sharma Post Graduate Institute of Medical Sciences (PGIMS), Rohtak, IND

**Keywords:** physical functional impairments, physical function, physical impairments, breast cancer survivors, breast cancer

## Abstract

Introduction and aim: Anti-cancer treatment imparts a variety of physical impairments that cause limitations in physical functioning among women with breast cancer. The aim of the study was to explore the opinions of healthcare professionals (HCPs) working with breast cancer patients on various aspects of physical functional impairments in breast cancer patients and survivors (BCP&S).

Methodology: The study was a cross-sectional survey. Taking into consideration the literature definition of ‘physical function’, its determinants, and literature published on relevant clinical factors in breast cancer, a survey questionnaire containing 29 questions was constructed. Thirty-seven HCPs, including physiotherapists, occupational therapists, and medical cancer experts, participated in the study. The participant's responses were obtained using a 5-point ‘Agreement’ Likert scale. Data analysis included a frequency table and the reliability test (Cronbach's alpha).

Results: The reliability of the questionnaire used in the survey was found to be acceptable (Cronbach’s alpha = 0.891). The majority of the participants were of the opinion that various parameters and determinants of ‘physical function’ get adversely affected in BCP&S, leading to limitations in the performance of activities of daily living (e.g., dressing and bathing), particularly in elderly and frail women. Participants agreed that such impairments in physical functioning affect social and role functioning and the overall quality of life (QoL) of women with breast cancer negatively.

Conclusion: This study found that various parameters and determinants of physical functioning are adversely affected in BCP&S, and physical functional impairments are prevalent in women with breast cancer, affecting their QoL negatively.

Implications for breast cancer patients: This study points out the need for long-term surveillance of BCP&S for physical functional limitations and a proactive treatment approach to prevent such limitations.

## Introduction

Breast cancer is the most common malignancy among women globally, and it is the leading cause of global cancer incidence [1]. From being fourth on the list of most common cancers in India during the 1990s, it has now become the first [2]. With advancements in diagnosis and the availability of treatment facilities, the survival time of breast cancer patients has significantly improved, but they still suffer from a variety of cancers and their treatment-related side effects involving multiple body tissues and systems, even after the completion of active treatment.

In literature [3], 'impairment' has been defined as a dysfunction or a significant structural abnormality in a specific body part or system. Breast cancer and its treatment-related side effects produce a variety of transient or prolonged physical impairments in the body, including peri-scar adherence, reduction in mobility and flexibility in the shoulder complex and chest, postural abnormalities, motor weakness and sensory impairments, upper limb lymphedema, cardiac and pulmonary dysfunctions, etc. [4-8]. These impairments, alone or in combination, contribute to limitations in physical functioning during or even after the completion of active treatment. Advanced age, the presence of co-morbidities, obesity, lower educational attainment, and smoking have been associated with a further decline in functional status in this patient population [9].

‘Physical function’ has been defined by Painter et al. [10] as 'the ability to perform the basic actions that are essential for maintaining independence and carrying out more complex activities'. An adequate level of physical functioning is essential to performing activities of daily living, for safe and independent living, and for participation in social, vocational, and recreational activities. Many determinants of physical function have been reported in the literature [10-11], including physical fitness level, disease-specific clinical factors, sensory factors, environmental factors, and behavioural factors. Many of these determinants of physical function have been found to be negatively affected in women with breast cancer. Tasmuth et al. [12] reported that 80% of 93 women who had undergone surgery for breast cancer had treatment-related symptoms, including pain, numbness, edema, strange sensations, and phantom sensations, and virtually all patients had symptoms in the ipsilateral arm one year after the surgery. Hidding et al. [13], in a systematic review of treatment-related impairments in the arm and shoulder in patients with breast cancer, reported that patients treated with axillary lymph node dissection had a higher risk of developing upper quarter impairments, including reduction in range of motion, strength, lymphoedema, and significant limitations in activities of daily living. They reported that radiotherapy and hormonal therapy were major risk factors for pain.

Studies [14] have reported that in cancer rehab publications with a focus on physical function, 'physical function' is predominantly measured using general health-related quality-of-life tools with 'physical function' as a subscale. Hence, cancer and its treatment-related physical functional problems are under-identified and under-treated.

Healthcare professionals (HCPs) working with breast cancer patients have comprehensive information and understanding of the physical impairments and physical functional limitations experienced by these patients during or after the completion of active treatment. Their opinions can be a valuable guide in understanding such limitations. However, to the best of our knowledge, no study has explored the opinion of HCPs working with breast cancer patients on various aspects of physical functional impairments in breast cancer patients and survivors (BCP&S).

Therefore, this study aimed to explore the opinions of healthcare professionals working with breast cancer patients on various aspects of physical functional impairments in breast cancer patients and survivors.

## Materials and methods

This study was an online cross-sectional survey conducted between January and September 2023. The ethical approval for the study was obtained from the Institutional Ethical Committee of Amity University, Noida (AUUP/IEC/DEC/2022/2). Participant-informed consent was obtained for the survey. Physiotherapists, occupational therapists, and medical cancer experts with more than three years of experience working with breast cancer patients were included in the study. Physiotherapists, occupational therapists, and medical cancer experts with less than three years of experience working with breast cancer patients were excluded. A convenience sampling method was used.

Questionnaire design

To design the survey questionnaire, a literature search was done to find out the definition, various aspects, and determinants of 'physical function' (definition and determinants described in an earlier section) [10,11]. Further, as per WHO’s ICF criteria [15,16], the following domains also need to be included in the functional assessment of an individual: body structures and body functions, activities and participation in life situations, and environmental factors as facilitators or barriers. Taking into consideration this literature definition of 'physical function' and its determinants as they relate to breast cancer, literature published on relevant clinical factors, and ICF criteria, a survey questionnaire was constructed. Two experts, including a senior physiotherapist and a medical cancer expert, reviewed and refined the survey questionnaire. Five practicing physiotherapists pilot-tested the questionnaire and further refined it. The final questionnaire ('Physical Function in Breast Cancer' questionnaire) consists of 28 questions related to various aspects of 'physical function' and its impairment in breast cancer patients and survivors, and one open-ended question inviting any other suggestion regarding physical function in breast cancer was developed, followed by the creation of a Google form. The questionnaire included questions pertaining to limitations experienced by breast cancer patients and survivors in performing basic activities of daily living (feeding, bathing, toileting, dressing, and transferring) and instrumental activities of daily living (shopping, doing household work, managing finances and transportation, cooking, lifting and carrying tasks, managing grooming needs, etc.). The questionnaire also included questions pertaining to physical fitness parameters in BCP&S (aerobic capacity, mobility and flexibility in the upper quadrant and hand grip strength, upper quadrant postural abnormalities, motor weakness in limbs, and sensory impairments in the body) and clinical factors associated with breast cancer treatment-related side effects that affect physical function directly or indirectly (pain, lymphoedema, chronic fatigue, impairment of memory and concentration, axillary web syndrome, painful phantom sensation, and impairment of balance). The questionnaire also had a question about the patient’s body image perception and the overall effect of physical functional limitations on social and role functioning, leisure activity time, and quality of life in BCP&S. The survey form included brief information about the survey and asked for participant information. The participants were requested to tick the appropriate response to each question on a 5-point 'agreement' Likert scale, with responses ranging from 'strongly disagree to strongly agree' (supplementary material). 

The reliability of the survey questionnaire was established. However, the validity of the questionnaire used in this survey was not established at this point, as this expert survey is part of another study on tool development, and the validity of the tool itself will be established at a later date. This study was written in accordance with the Checklist for Reporting of Survey Studies (CROSS) [17].

Procedure

Qualified physiotherapists, occupational therapists, and medical cancer experts working with breast cancer patients in various hospitals across India were approached directly or by snowball sampling. An invitation to participate in the study, along with an anonymous survey link, was sent to 49 professionals through social media platforms or e-mail, and they were requested to share the survey link in their social media groups as well. The study's participation was voluntary, and multiple participations by the participants were not allowed. The participants were encouraged to ask the principal investigator any questions about the study. Forty-two participants consented to participate in the study and filled out the survey form. Out of 42 participants who responded, five responses were not included in the final data analysis, as four of them had less than three years of experience working with breast cancer patients. One participant did not respond to five of the total questions and also did not respond to further communication. Out of 37 participants included, five had not reported answers to one question each and were telephonically contacted to obtain their responses to missed questions. All these five participants, when enquired about the reason for the missing question, informed that the missing of question was not deliberate. A total of 37 participant responses were included in the final data analysis. The 'strongly agree' and 'agree' response choices were considered positive responses, and the 'strongly disagree' or 'disagree' response choices were considered negative responses. The following data were collected: gender, professional qualification, years of experience working with breast cancer patients, and responses to survey questions.

The statistical analysis was carried out using IBM SPSS (Statistical Package for Social Sciences) version 20 (IBM Corp., Armonk, NY). The analysis includes a frequency table and the reliability test, Cronbach's alpha.

## Results

The following data were statistically analysed using IBM SPSS statistical version 20: gender, professional qualification, years of experience working with breast cancer patients, responses to survey questions, and questionnaire reliability.

A total of 37 participants were included in the study. Gender-wise, 15 (40.5%) participants were females and 22 (59.5%) were males. Profession-wise, 20 (54.1%) participants were physiotherapists, 8 (21.6%) were occupational therapists, and 9 (24.3%) were medical cancer experts. Twenty-one (56.8%) participants had 3-10 years, 12 (32.4%) had 11-20 years, and 4 (10.8%) had 21-30 years of experience working with breast cancer patients (Table 1).

**Table 1:** Participant (n=37) demographics.

S.No.	Participant characteristics		Number of participants N (%)
1.	Gender	Female	15 (40.5 %)
		Male	22 (59.5 %)
2.	Profession	Physiotherapist	20 (54.1 %)
		Occupational therapist	8 (21.6 %)
		Medical cancer experts	9 (24.3 %)
3.	Experience (years)	3–10 years	21 (56.8 %)
		11–20 years	12 (32.4 %)
		21–30 years	4 (10.8%)

The reliability of the 'Physical Function in Breast Cancer Questionnaire' was found to be acceptable (Cronbach’s alpha = 0.891). ‘Strongly agree’ and ‘agree’ response choices were considered positive responses, and strongly disagree’ or ‘disagree’ response choices were considered negative responses. Further, 'strongly agree’ and ‘agree’ responses were taken together as ‘agreed’ responses, and ‘strongly disagree’ and ‘disagree’ responses were taken together as ‘disagreed’ responses, and ‘neither agree nor disagree’ response was taken as ‘neutral response’. The responses obtained from healthcare professionals are depicted in Table 2.

**Table 2:** Participant (n=37) responses.

S.No.	Question	Participant responses = N (valid %)		
		Agreed (‘Agree’ and ‘Strongly agree’)	Neutral nor (‘Neither agree' or 'disagree')	Disagreed (‘Disagree’ and ‘Strongly disagree’)
1.	Do some breast cancer patients/survivors, particularly the elderly and frail, experience difficulty feeding, bathing, and dressing themselves?	30 (81.1%)	2 (5.4%)	5 (13.5%)
2.	Do some breast cancer patients/survivors, particularly the elderly and frail, experience difficulty in transferring themselves (within bed, from bed to chair or car etc.)?	19 (51.4%)	7 (18.9%)	11 (29.7%)
3.	Do some breast cancer patients/survivors, particularly elderly and frail, experience difficulty in managing their toileting needs (transfer to and from seat, washing and adjusting clothes)?	27 (73.0%)	3 (8.1%)	7 (18.9%)
4.	Do some breast cancer patients/survivors experience difficulty in managing their grooming needs (e.g., combing hair)?	31 (83.8%)	5 (13.5%)	1 (2.7%)
5.	Do some breast cancer patients/survivors experience reduction in mobility and flexibility in upper quadrant (e.g., stiff shoulder joint, reduction in chest mobility)?	35 (94.6 %)	1 (2.7%)	1 (2.7%)
6.	Do many breast cancer patients/survivors develop postural abnormalities in upper quarter (e.g., rounded shoulders) during or post cancer treatment?	32 (86.5%)	3 (8.1%)	2 (5.4%)
7.	Do some breast cancer patients/survivors experience limitation in doing light household work (e.g., dusting, shifting, and carrying light objects etc.)?	17 (45.9%)	13 (35.1%)	7 (18.9%)
8.	Do many breast cancer patients/survivors experience difficulty in doing heavy household work (cleaning floors, shifting heavy objects like sofa)?	35 (94.6%)	1 (2.7%)	1 (2.7%)
9.	Do many breast cancer patients/survivors experience difficulty in performing lifting and carrying tasks (e.g., carrying shopping bag, laptop bag, purse) on affected side shoulder or arm?	31 (83.8%)	4 (10.8%)	2 (5.4%)
10.	Do many breast cancer patients/survivors, experience limitation in performing Instrumental activities of daily living (shopping, managing finances, medication and transportation related needs, using common gadgets which they used before illness, etc.) during or post-cancer treatment?	18 (48.6%)	5 (13.5%)	14 (37.8%)
11.	Do some breast cancer patients/survivors experience difficulty in preparing balanced meal?	14 (37.8%)	15 (40.5%)	8 (21.6%)
12.	Do many breast cancer patients/survivors are significantly limited in doing day‑to‑day activities due to pain?	28 (75.7%)	4 (10.8%)	5 (13.5%)
13.	Do many breast cancer patients/survivors complain of poor quality of sleep due to pain?	24 (64.9%)	7 (18.9%)	6 (16.2%)
14.	Do breast cancer patients/survivors experience more frequent falls or fear of fall in day-to-day activities?	8 (21.6%)	7 (18.9%)	22 (59.5%)
15.	Do some breast cancer patients/survivors experience chronic fatigue in day-to-day life?	28 (75.7%)	9 (24.3%)	-
16.	Does in some breast cancer patients/survivors, chronic fatigue limits physical, social, role functioning?	30 (81.1%)	5 (13.5%)	2 (5.4%)
17.	Do some breast cancer patients/survivors experience difficulty in performing prolonged physical activity (e.g., taking long walks)?	24 (64.9%)	6 (16.2%)	7 (18.9%)
18.	Do breast cancer patients/survivors experience remarkable difficulty in using arm with lymphoedema?	34 (91.9%)	2 (5.4%)	1 (2.7%)
19.	Do breast cancer patients/survivors have reduced Aerobic Capacity?	22 (59.5%)	9 (24.3%)	6 (16.2%)
20.	During or after breast cancer treatment, do some patients/survivors develop impairment of memory and concentration which adversely affects their performance in day-to-day life?	5 (13.5%)	15 (40.5%)	17 (45.9%)
21.	Does Axillary Web syndrome cause remarkable limitation in using arm in day-to-day activities?	32 (86.5%)	5 (13.5%)	-
22.	Do many breast cancer patients/survivors experience development of motor weakness (reduction in muscle strength and endurance) in limbs particularly in affected side upper limb?	29 (78.4%)	4 (10.8%)	4 (10.8%)
23.	Do some breast cancer patients/survivors experience sensory impairments (numbness, paresthesia, etc.) in limbs, which limits the use of affected limb in functional activities?	20 (54.1%)	10 (27.0%)	7 (18.9%)
24.	Do breast cancer patients/survivors experience reduction in hand grip strength leading to impaired hand function (difficulty in writing/ typing/painting/ weaving/ knitting/ doing handicraft work etc.)?	21 (56.8%)	8 (21.6%)	8 (21.6%)
25.	Do many breast cancer patients or survivors feel conscious about their physical appearance which affects their performance altogether?	32 (86.5%)	3 (8.1%)	2 (5.4%)
26.	Do some breast cancer patients/survivors experience painful phantom sensation which affects their overall performance negatively?	16 (43.2%)	15 (40.5%)	6 (16.2%)
27.	Do physical functional limitations affect quality of life of a breast cancer patients/survivors negatively?	30 (81.1%)	3 (8.1%)	4 (10.8%)
28.	Do physical functional limitations lead to reduction in Leisure activity time of a breast cancer patient/survivor?	26 (70.3%)	7 (18.9%)	4 (10.8%)

The majority of the participants ‘agreed’ that many BCP&S develop reductions in physical fitness-related parameters. Participants agreed that many BCP&S develop motor weakness in the affected side arm 29 (78.4%), reduction in mobility and flexibility in the shoulder complex and chest 35 (94.6%), and postural abnormalities in the upper quadrant 32 (86.5%). However, only 22 (59.5%) ‘agreed’ about a reduction in aerobic capacity, and 21 (56.8%) ‘agreed’ about a reduction in hand grip strength on the affected sidearm.

The majority of participants ‘agreed’ that many clinical factors associated with breast cancer or its treatment-related side effects, including pain, lymphoedema 34 (91.9% agreed), chronic fatigue 30 (81.1% agreed), and axillary web syndrome 32 (86.5% agreed), cause a reduction in active usage of arm in day-to-day life. Twenty-eight (75.7%) participants ‘agreed’ that pain produces significant limitations in doing day-to-day activities, and 24 (64.9%) ‘agreed’ that it leads to poor quality of sleep among breast cancer patients and survivors. However, 22 (59.5%) participants ‘disagreed’ regarding balance-related problems leading to increased frequency of falls or fear of falling among BCP&S.

Thirty (81.1%) of the participants ‘agreed’ that many BCP&S, particularly the elderly and frail, experience short- or long-term difficulty performing basic activities of daily living, including feeding, bathing, dressing themselves, and managing toileting needs. Fourteen (37.8%) participants ‘disagreed’ and 5 (13.5%) participants had a ‘neutral’ response about the difficulty experienced by breast cancer patients in performing instrumental activities of daily living, including shopping for personal and family requirements, managing finances, medication and transportation needs, and preparing a balanced meal.

Thirty-two (86.5%) participants were of the opinion that BCP&S who had undergone mastectomy feel conscious of their physical appearance while working at the workplace or at home, which negatively impacts their performance altogether. The majority of participants ‘agreed’ that a decline in physical functioning also reduces leisure activity time, social and role functioning levels, and quality of life in BCP&S.

The open-ended question, which invited further suggestions regarding physical functioning in BCP&S, received no significant responses, with the exception that adequate rehabilitation can significantly limit the decline in physical functioning levels in this patient population.

## Discussion

Few studies have been conducted to evaluate physical functional impairments in BCP&S. To the best of our knowledge, no study has been conducted on healthcare professionals working with breast cancer patients to evaluate their perspective on various aspects of physical functional limitations in BCP&S. Therefore, this study aimed to explore the opinions of healthcare professionals working with breast cancer patients on various aspects of physical functional impairments in breast cancer patients and survivors.

Opinions of physiotherapists, occupational therapists, and medical cancer experts on physical functional limitations in BCP&S were obtained in this study as these professionals, educationally and clinically, examined and treated such limitations in this patient population. Social media platforms have helped to reduce various barriers to conducting studies, such as distance, time, and cost. Therefore, a social media network was used in this survey to circulate survey links among professionals.

The results of the study revealed that a significant number of participants were of the opinion that many BCP&S experience reductions in physical fitness-related parameters, including reductions in mobility and flexibility in the upper quadrant, upper quadrant postural abnormalities, motor weakness and sensory impairments, and reductions in aerobic capacity. Oncology-directed treatments such as surgery, chemotherapy, as well as radiation therapy to the chest wall produce various side effects and impairments in the body. Physical impairments in the upper quarter are quite prevalent in BCP&S. Further, during or after the completion of active treatment, many BCP&S self-restrict their activity level, abandoning some activities or changing the way they are performed due to fear of causing injury to the affected sidearm. This conscious reduction in the usage of the arm causes a secondary decrease in flexibility, strength, and endurance in the arm and shoulder complex. Radiation therapy to the chest wall also produces cardio-pulmonary dysfunction in the short and long term, reducing aerobic capacity, exercise tolerance, and endurance in this patient population. A reduction in all these parameters of physical fitness causes a reduction in the level of physical functioning in BCP&S. The findings of this survey are consistent with the findings of Levangie et al. [18], who evaluated the long-term effect of breast cancer treatment on upper quarter function in breast cancer survivors, as reported by physical therapists who were either breast cancer survivors themselves or who specialised in the treatment of individual post-breast cancer patients. The authors had reported a high prevalence of breast cancer-related upper quadrant dysfunction, including pain, decreased range of motion and muscle strength, lymphoedema, cording, numbness, etc., in breast cancer survivors, which caused limitations in physical activity and participation. 

The majority of study participants were of the opinion that clinical factors associated with breast cancer treatment-related side effects, including pain, lymphoedema, chronic fatigue, axillary web syndrome, and impairment of balance, reduce the physical functioning levels in the day-to-day lives of many breast cancer patients and survivors. A great proportion of healthcare professionals agreed that many BCP&S, particularly the elderly and frail, experience short- or long-term limitations in performing basic and instrumental activities of daily living. Due to pain, reduced flexibility, lymphoedema, chronic fatigue and frailty, and self-restriction of activities for fear of causing injury to the affected side arm, many women, particularly the elderly, tend to reduce their activity level, which is reflected in the performance of basic and instrumental activities of daily living as well. Karki et al. [19], in a prospective survey study on 110 breast cancer patients, reported that at 6 and 12 months post-operation, scar tightness, axillary oedema, neck arm pain, and difficulty in lifting, carrying, and reaching out activities were prevalent in breast cancer patients. The authors also reported frequent and constant activity limitations and participation restrictions at work, at home, and in leisure activities, even 12 months after the operation. 

The majority of the healthcare professionals (n = 32, 86.5%) were also of the opinion that negative body image perceptions and consciousness about physical appearance in patients who have undergone a mastectomy significantly affect their overall performance. Self-consciousness, worries, and difficulties experienced by some patients and survivors about the new prosthesis and clothing distract them from giving their free performance at the workplace or home. In the literature, studies [20,21] have also reported the adverse impact of negative body image perception and consciousness about physical appearance on function in breast cancer survivors.

The majority of the participants agreed that physical functional limitations have a negative effect on the social, role functioning, leisure activity time, and quality of life of many breast cancer patients and survivors.

Our survey results support and complement the findings of other investigators who conducted studies on BCP&S to evaluate impairments in physical functioning in this patient population. Braithwaite et al. [22] reported the presence of at least one physical functional limitation in 39% of study participants (aged 21-79 years) in a cohort of 2202 women with breast cancer. Satarino et al. [23] also reported a significant difference in physical functioning levels among women with breast cancer aged 55 years and older when compared to controls of the same age. Kroenke et al. [24] also reported that, as compared to women without breast cancer, women with breast cancer experienced a significant decline in physical function, vitality, and emotional function in all age groups.

Limitations

The study had small sample size. The validity of the questionnaire was not established.

Future research

In the future, more such multicentre surveys involving a higher number of professionals across a wider geographical area can be conducted to better understand the physical function-related limitations experienced by breast cancer patients and survivors so that further rehabilitation interventions to address such limitations can be explored.

## Conclusions

This study found that, as per health care professionals working with breast cancer patients, physical functional impairments are prevalent in women with breast cancer and affect their quality of life negatively. This study points out the need for long-term surveillance of breast cancer patients and survivors for physical functional limitations using valid and reliable physical functional assessment tools. Proactive treatment for the prevention and treatment of such physical and functional limitations should be provided to women with breast cancer.

## Appendices

Supplementary material

Questionnaire Link: https://docs.google.com/forms/d/e/1FAIpQLSdeXl3mCIkchs090Tn_PsoChARVXGWMMFpVuGu1lWg_aVunEQ/viewform?usp=sf_link
